# Reliability, reproducibility and validity of dynamic cerebral autoregulation in a large cohort with transient ischaemic attack or minor stroke

**DOI:** 10.1088/1361-6579/abad49

**Published:** 2020-10-06

**Authors:** Yun-Kai Lee, Peter M. Rothwell, Stephen J. Payne, Alastair J.S. Webb

**Affiliations:** 1 Institute of Biomedical Engineering, Department of Engineering Science, University of Oxford; 2 Wolfson Centre for Prevention of Stroke and Dementia, Nuffield Department of Clinical Neurosciences, John Radcliffe Hospital, University of Oxford

**Keywords:** Cerebral Autoregulation, Reliability, Reproducibility, Stroke, Transient Ischaemic Attack

## Abstract

**Objective:**

Cerebral autoregulation (CA) is critical to maintenance of cerebral perfusion but its relevance to the risk of stroke and dementia has been under-studied due to small study sizes and a lack of consensus as to the optimal method of measurement. We determined the reliability and reproducibility of multiple CA indices and the effect of intensive data-processing in a large population with transient ischaemic attack or minor stroke.

**Approach:**

Consecutive, consenting patients in the population-based OXVASC (Oxford Vascular Study) Phenotyped cohort underwent up to 10-min supine continuous blood pressure monitoring (Finometer) with bilateral middle cerebral artery (MCA) transcranial ultrasound (DWL-Dopplerbox). Un-processed waveforms (Un-A) were median-filtered, systematically reviewed, artefacts corrected and their quality blindly graded (optimal (A) to worst (E)). CA metrics were derived in time-domain (autoregulatory index (ARI), Pearson’s Mx, Sx, Dx) and in very-low (VLF) and low-frequency (LF) domains (WPS-SI: wavelet phase synchronisation, transfer function analysis), stratified by recording quality. Reliability and reproducibility (Cronbach’s Alpha) were determined comparing MCA sides and the first vs. second 5-min of monitoring.

**Main results:**

In 453 patients, following manual data-cleaning, there was good reliability of indices when comparing MCA sides (Mx: 0.77; WPS-SI-VLF: 0.85; WPS-SI-LF 0.84), or repeated five minute epochs (Mx: 0.57; WPS-SI-VLF: 0.69; WPS-SI-LF 0.90), with persistently good reliability between sides even in lower quality Groups (Group D: Mx: 0.79; WPS-SI-VLF: 0.92; WPS-SI-LF: 0.91). Reliability was greatest for Pearson’s Mx and wavelet synchronisation index, with reasonable reliability of transfer function analyses, but ARI was prone to occasional, potentially defective, extreme estimates (Left vs right MCA: 0.68).

**Significance:**

Resting-state measures of CA were valid, reproducible and robust to moderate noise, but require careful data-processing. Mx and wavelet synchronisation index were the most reliable indices for determining the prognostic value of CA in large epidemiological cohorts and its potential as a treatment target.

## Introduction

Cerebral autoregulation (CA) is a vital physiological mechanism to maintain constant cerebral perfusion despite changes in systemic blood pressure (BP) (Xiong et al. 2017; Donnelly et al. 2016). Dynamic CA in response to rapid BP changes can be estimated from direct physiological challenges or from resting-state fluctuations in BP, with previous studies showing a symmetry and a significant correlation between CA indices derived from the contralateral middle cerebral arteries (MCAs) (Schmidt et al. 2003). CA is impaired in severe traumatic brain injury (Liu et al. 2017; Czosnyka et al. 2001; Schmidt et al. 2016), intracerebral haemorrhage (Lee et al. 2017; Ma et al. 2016; Oeinck et al. 2013), subarachnoid haemorrhage (Budohoski et al. 2012, 2013), and major ischaemic stroke (Reinhard et al. 2012; Chi et al. 2018). However, its role in predicting the risk of recurrent events is unclear. Specifically, dynamic CA in transient ischaemic attack (TIA) or minor stroke has not been adequately investigated. Studies that have assessed CA in TIA have reported a preserved CA in bilateral MCAs acutely and sub-acutely, with impaired CA in major stroke (Allan et al. 2015; Atkins et al. 2010), but the smaller study sizes may limit the understanding of its relevance to the risk of stroke and its importance as a risk factor and target for treatment.

Despite the potential prognostic importance, there is no current consensus as to the optimal method of assessing CA, with methods available using either CT perfusion, MR imaging, or cerebral blood flow velocity (CBFV) on transcranial ultrasound (Brodie et al. 2009; Elting et al. 2014; Chi et al. 2018), with no standardised analytic approaches resulting in numerous metrics of CA (Sanders et al. 2018, 2019). Furthermore, heterogeneity between patient groups in multiple small studies results in high variability in reported CA metrics between studies (Sanders et al. 2018, 2019). Such limitations underlie this lack of consensus (Brodie et al. 2009; Elting et al. 2014; Chi et al. 2018), preventing the determination of the prognostic value of CA and applicability in clinical practice (Sanders et al. 2018, 2019).

Previous reports have investigated the reproducibility of CA between different visits or recording lengths in limited populations, assessing specific indices (Brodie et al. 2009; Elting et al. 2014; Chi et al. 2018) or using simulated CBFV signals (Sanders et al. 2018, 2019; Liu et al. 2020). Few studies report reliability and reproducibility of multiple CA metrics derived from actual beat-to-beat average of signals. This limited evidence suggests that poor reproducibility of signals reflects either physiological variability or non-stationarity of signals (Sanders et al. 2019), but short-term reproducibility has not been systematically assessed in a clinical population or a population of adequate size. Furthermore, the effect of noise and the impact of manual data-cleaning, has not been determined.

Therefore, in a population-based cohort of patients with TIA or minor stroke at increased risk of future cardiovascular events and dementia, we determined reliability and reproducibility of different indices of CA between cerebral hemispheres and between separate periods of recording, and the effect of intensive data cleaning, to identify their suitability for assessment of prognostic significance.

## Materials and Methods

### Study Population and Research Ethics Approval

Consecutive patients were recruited between September 2010 and September 2017 from the Oxford Vascular Study (OXVASC) TIA and minor stroke clinic (Rothwell et al. 2004). The OXVASC population consists of more than 92,000 individuals registered with 100 primary-care physicians in Oxfordshire, United Kingdom (Rothwell et al. 2004; Webb et al. 2012, 2018). All consenting patients underwent a standardised medical history and examination, ECG, blood tests and a stroke protocol magnetic resonance brain imaging and contrast-enhanced magnetic resonance angiography (or CT-brain and carotid Doppler ultrasound or CT angiogram), an echocardiogram and 5-day ambulatory cardiac monitoring. All patients were reviewed by a study physician, the diagnosis verified by the senior study neurologist (P.M.R), the aetiology determined by a panel of stroke neurologists and are followed-up face-to-face for up to 10 years. Consenting, consecutive patients underwent a physiological assessment at the 1-month follow-up visit. Participants were excluded from this analysis if they were <18 years of age, cognitively impaired (Mini-Mental State Examination <23), pregnant; had atrial fibrillation, active cancer, autonomic failure, a recent myocardial infarction, unstable angina, heart failure (New York Heart Association, 3–4 or ejection fraction, <40%) or untreated bilateral carotid stenosis (>70%). OXVASC is approved by the Oxfordshire Research Ethics Committee (Rothwell et al. 2004; Webb et al. 2012, 2018, 2019).

### Data Acquisition and Pre-processing

Patients were tested at the ascertainment or 1-month clinic visit in a quiet, dimly-lit, temperature-controlled room (21–23°C). Continuous 3-lead electrocardiogram (ECG) and non-invasive finger arterial blood pressure (ABP) were acquired (Finometer MIDI, Finapres Medical Systems) via a Powerlab 8/35 (ADInstruments). Cerebral blood flow velocity (CBFV) from bilateral middle cerebral arteries (MCAs) was simultaneously measured using transcranial Doppler (TCD) ultrasound (DWL Dopplerbox; Compumedics DWL, Singen, Germany), identifying the optimal site of insonation with a handheld 2-MHz probe at the temporal bone window before recording continuous waveforms with 2-MHz monitoring probes attached to a headset worn by the participant to reduce motion, recording the highest velocity trace between 50-55 mm depth, where feasible (Rothwell et al. 2004; Webb et al. 2012, 2018, 2019). BP and ECG waveforms were acquired at 200 Hz and TCD at 100Hz, during up to 10 minutes of supine rest. All consecutive beat-to-beat averages of signals of both ABP and CBFV from bilateral MCAs were median filtered (7 data points), with automatic detection and linear interpolation of ectopic beats, and were then visually reviewed by an experienced operator (A.J.S.W.) for blinded quality assessment with additional linear interpolation of artefacts due to motion (Rothwell et al. 2004; Webb et al. 2012, 2018, 2019). All records were quantified into: 3 (optimal), 2 (adequate quality for analysis), 1 (unusable, severe artefact) and 0 (no data) according to their signal quality. We then stratified all patients into Groups A (both ABP and CBFV=3), B (CBFV=2 and ABP=3), C (ABP=2 and CBFV=3), D (both ABP and CBFV=2), and E (ABP=3 or 2; CBFV = 1 or 0) according to the quality of each signal (Webb et al. 2012, 2018, 2019).

To determine the effect of manual data cleaning and signal quality, CA indices were derived and compared for un-processed data in the best quality group (labelled as Un-A) compared to cleaned data (Group A–E). To assess the trend of the data quality from the optimal to the worst group, the variability of the mean ABP (MABP) and bilateral CBFV (MFv) signals in each quality group was calculated as the coefficient of variation. Dynamic CA of all patients in each group was then estimated.

### Derivation of Indices of Dynamic Cerebral Autoregulation

All consecutive beat-to-beat signals were de-meaned and de-trended by linear regression. Dynamic CA was quantified by indices derived in both time- and frequency-domains. In the time-domain, autoregulatory index (ARI) and Pearson’s correlation coefficient-based parameters of mean, systolic, and diastolic flow indices (Mx, Sx, and Dx, respectively) were calculated (Mahdi et al. 2017a). For the indices derived from the frequency-domain, transfer function analysis (Claassen et al. 2015) (TFA) and wavelet phase synchronisation (WPS) algorithms were used to derive the TFA-based gain, phase, and coherence (Claassen et al. 2015) and WPS-based phase and synchronisation index (SI) (Latka et al. 2005; Peng et al. 2010) respectively. We determined the mean values of indices in very-low-frequency (VLF, [0.02 – 0.07 Hz]) and low-frequency (LF, [0.07 – 0.2 Hz]) as the most physiologically relevant frequency regions of CA (Claassen et al. 2015). Details of calculations of all CA metrics are described in full in the Supplemental Materials (Mahdi et al. 2017a; Claassen et al. 2015; Latka et al. 2005; Peng et al. 2010).

### Statistical Analysis

The distributions, associations, reliability, and reproducibility of CA indices were investigated. We estimated the variability of signals in each quality group (coefficient of variations (CV = (SD/Mean) × 100%)) and compared differences in CV% of both MABP and MFv of data sets before and after pre-processing (Un-A vs. Group A, paired sample t-test) and across quality groups A-E (analysis of variance (ANOVA); test for linear trend). We compared the demographic characteristics of recruited patients across groups A-E by using the one-way ANOVA for continuous data and the ordinal Chi-square (X^2^) test for categorical variables.

Distributions and associations were analysed by histograms with normality tests (Kolmogorov-Smirnov test and the Shapiro-Wilk test if the sample size of the group < 40) and linear regression respectively. Comparisons of CA indices between the first (1-5 min) and the second (6-10 min) recording epochs and with the entire 10-min recordings were conducted using non-parametric Friedman’s test, followed by Dunn’s multiple comparisons post-hoc analysis.

We assessed the reliability between right and left MCA in each quality group and the reproducibility between 5-min epochs in Un-A and Group A by Cronbach’s Alpha (Cronbach, 1951). Statistical comparisons between Alpha coefficients were conducted using the web interface and R-package ‘*cocron’* (Diedenhofen and Musch (2016)), where Group E was excluded from statistical comparisons due to poor reliability.

For all analyses, a *p*-value <0.05 was considered to be statistically significant. All analysis was performed in Microsoft Excel, R, Matlab 2017a, and GraphPad Prism 8 software.

## Results

### Characteristics of Study Population

453 of 656 consenting participants with adequate bone windows were recruited (table 1), with reasons for exclusion described in the study flowchart (supplementary figure I). After manual data pre-processing, the variability of signals reduced, with a significant reduction in MFv in LMCA (table 2; Un-A vs. Group A: p = 0.069 for CV% of MABP; and p = 0.13 and p <0.0001 for CV% of MFv in R- and LMCA, respectively). For the pre-processed Groups A – E, the proportion of problematic and un-usable recordings and variability of measures was higher in the worse quality groups (*p-trend* <0.0001 for CV% of MABP; and *p-trend* <0.0001 and <0.05 for CV% of MFv in R- and LMCA, respectively; table 2 and supplementary figure II).

### Comparisons of Dynamic CA Indices

**Figure 1:**
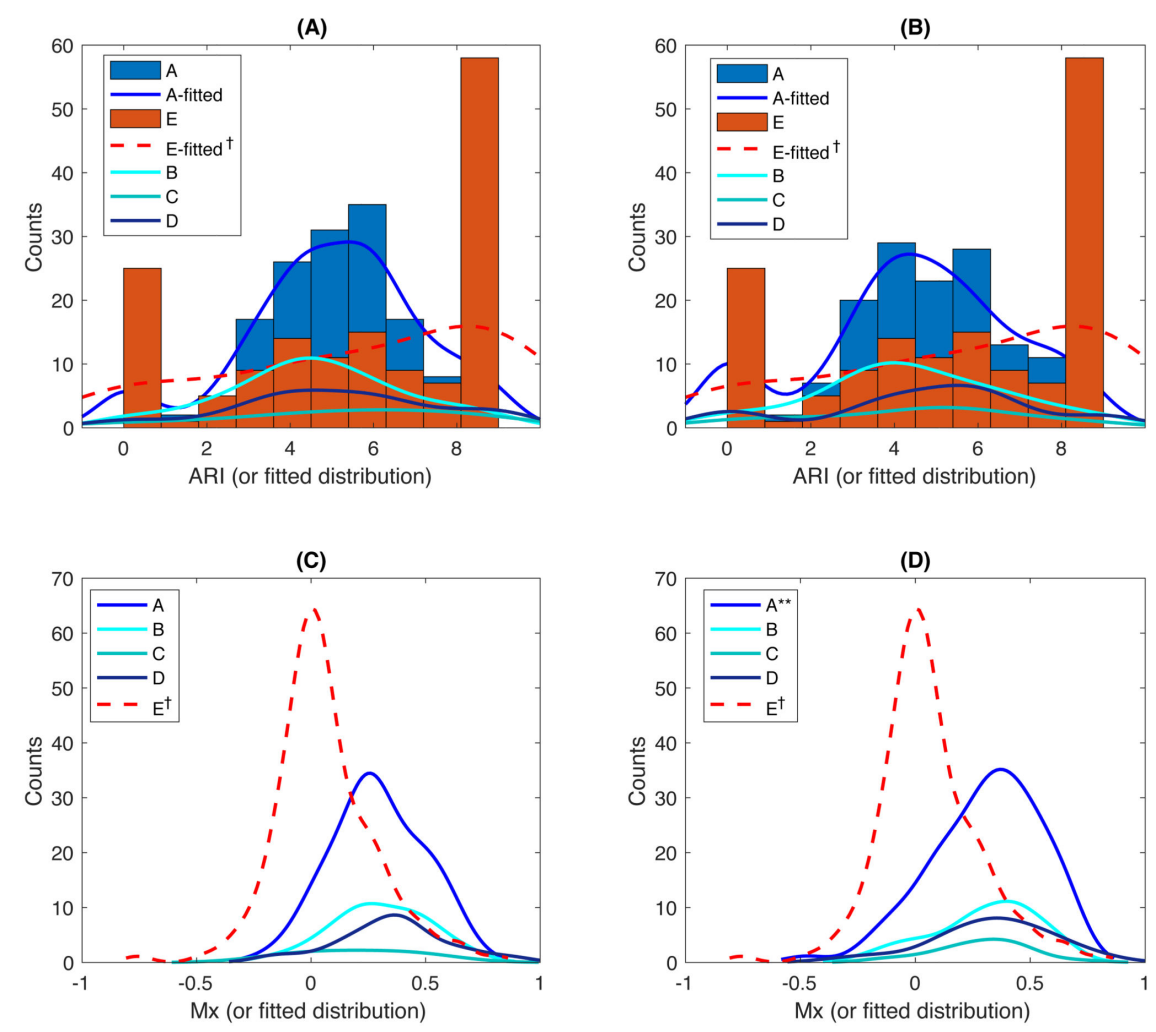
The distribution of ARI and Mx indices on both sides of MCA from Group A – E(no data side). (A) and (B) show the ARI index in R- and LMCA; (C) and (D) are Mx in R-and LMCA, respectively. **, p <0.001 and †, p <0.0001 by normality tests.

**Figure 2:**
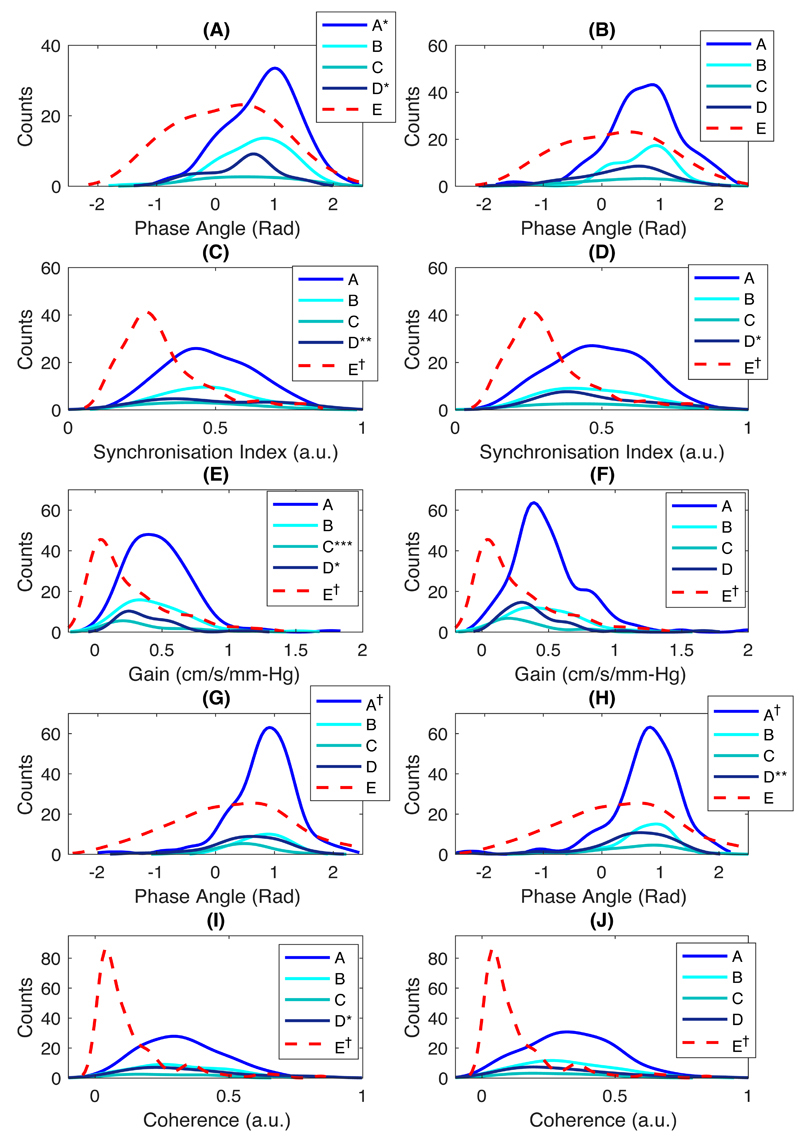
Distributions of frequency-domain indices in VLF band in both sides of MCA. (A – B) WPS-Phase; (C – D) WPS-SI; (E – F) TFA-based gain; (G – H) TFA-Phase; and (I – J) TFA-Coherence in R- and LMCA, respectively. *, p <0.05; **, p <0.01; ***, p <0.001; †, p <0.0001.

**Figure 3:**
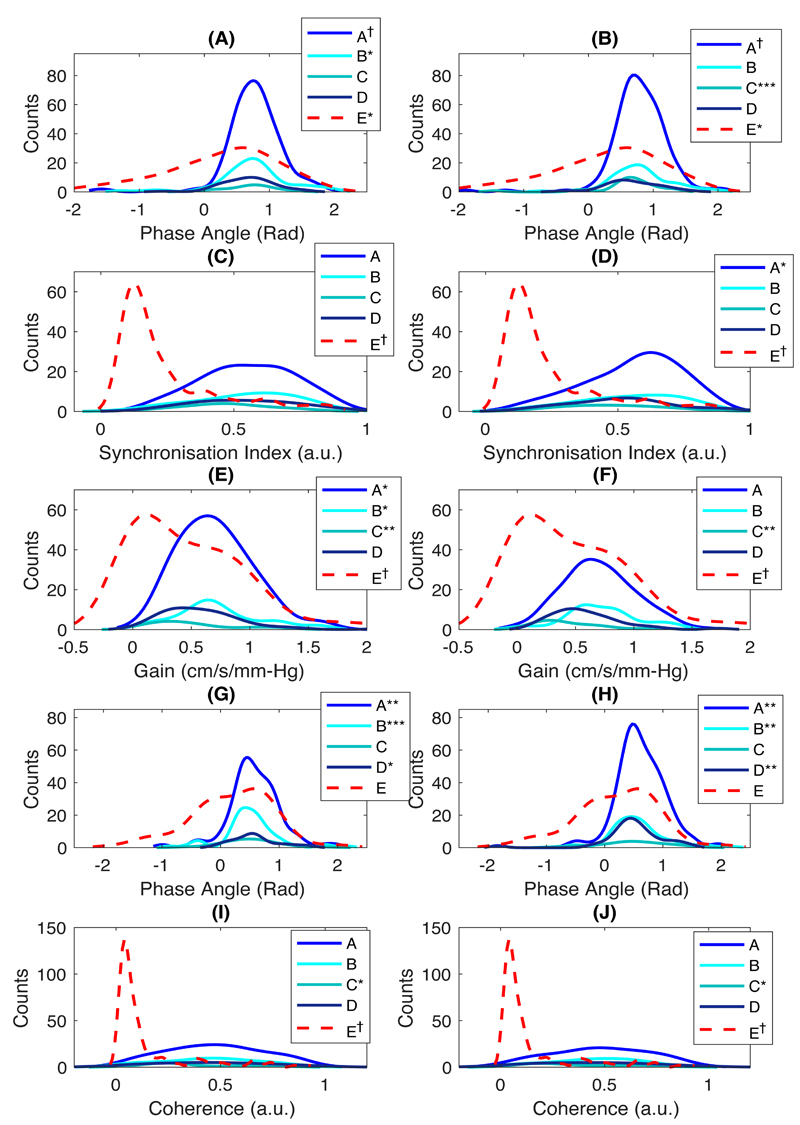
Distributions of frequency-domain indices in LF band in both sides of MCA. (A – B) WPS-Phase; (C – D) WPS-SI; (E – F) TFA-based gain; (G – H) TFA-Phase; (I – J) TFA-Coherence in R-and LMCA, respectively. *, p <0.05; **, p <0.01; ***, p <0.001; †, p <0.0001.

ARI was particularly sensitive to noise and artefact, with a higher proportion of artefactual extreme values of 0 and 9 in lower quality groups, resulting in a non-normal distribution (figure 1 (a – b)), whilst Pearson’s flow indices and most frequency-domain indices, despite being normally distributed, tended to zero (figure 1 (c – d); Sx and Dx behaved similarly to Mx (supplementary figure III); and figures 2 – 3). However, after exclusion of artefactual ARI values of 0 and 9, ARI in both MCAs were normally distributed (p >0.05 in Groups A – E by Kolmogorov-Smirnov test).

Significantly negative associations between ARI and Mx and the slopes of regression lines fell with lower quality data, with a consistent trend in both sides of MCA (Group A-RMCA vs. E-No data side: r^2^ = 0.49; p <0.0001; β = -0.067 vs. r^2^ = 0.0013; p = 0.17; β = -0.008; supplementary table I). Similarly, upon removing the extreme values of ARI, the negative associations became significant, even in lower quality groups. Overall, the slope of the regression lines in lower quality groups was flatter when compared to the optimal quality Group A (Group A-RMCA vs. E-No data side: r^2^ = 0.55; p <0.0001; β = -0.09 vs. r^2^ = 0.07; p = 0.001; β = -0.025; supplementary table I).

There were significantly negative associations between Mx and Phase in VLF (Mx and TFA-Phase: RMCA-β = -0.52; and Mx and WPS-Phase: RMCA-β = -0.52), whilst associations between Mx and TFA-coherence (RMCA-β = 0.40 and 0.27 in VLF and LF, respectively) and WPS-SI (RMCA-β = 0.32 and 0.28 in VLF and LF, respectively) were significantly positive (supplementary table II).

### Reliability between left and right MCA recordings

**Figure 4:**
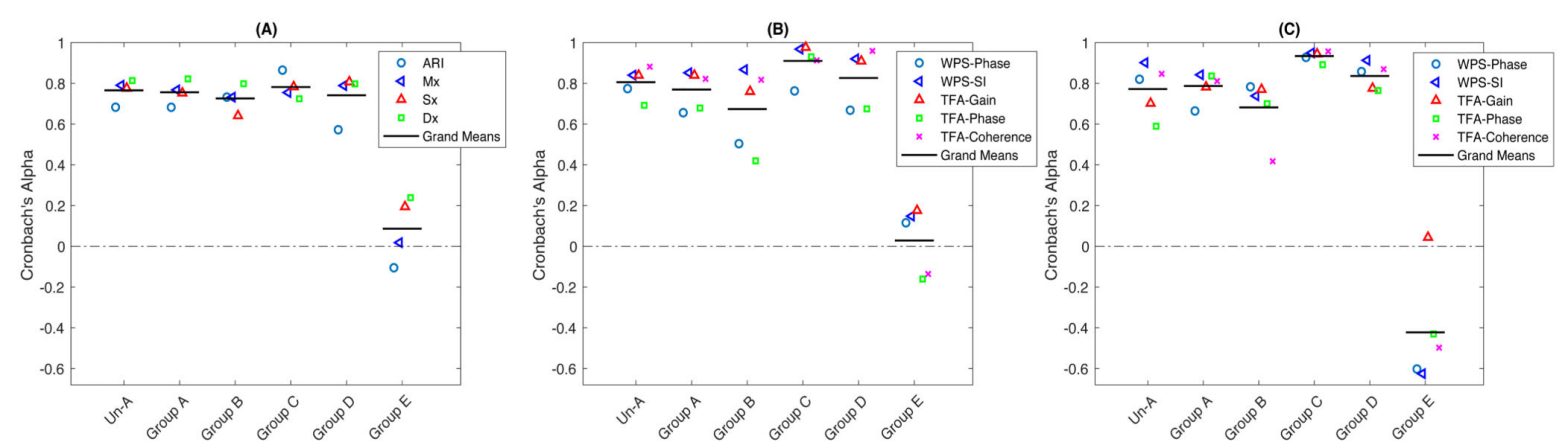
The reliability between right and left MCA from the optimal to the worst quality group. (A) time-domain indices; and (B) and (C) are frequency-domain indices in VLF and LF bands respectively. Two patients with only left-sided window were not included in the reliability analysis.

After manual data-cleaning, reliability of CA between MCA sides remained persistent, with an increased reliability of indices of Dx, WPS-SI in VLF, and TFA-Gain and TFA-Phase in LF. There was an increased mean reliability across all metrics after pre-processing in frequency domain indices in LF band (figure 4 (c) and table 3). Most indices persisted with a similar reliability value after pre-processing, whilst reliability of TFA-Phase in LF band increased significantly (Un-A vs. Group A: 0.59 vs. 0.84; p <0.001; table 3) but WPS-Phase in LF showed an opposite direction of significant changes (Un-A vs. Group A: 0.82 vs. 0.66; p <0.05). Across all quality groups, the lowest quality Group E had the poorest reliability in both time- and frequency-domain metrics (table 3 and figure 4), with minimal reliability between sides. Group E was therefore excluded from statistical comparisons between indices.

For time-domain indices, ARI had the lowest reliability even in the optimal Group A but the reliability of the Pearson’s coefficient indices of Mx, Sx, and Dx was preserved even in the lower quality Group D (all reliability ≥0.79; table 3 and figure 4 (a)). Following data-cleaning, reliability of all indices was consistent across groups (A – D), after exclusion of the worst quality data (Group E).

For CA metrics in the VLF band, both WPS-Phase and TFA-Phase had the lowest reliability across groups (A-D). The other indices including WPS-SI, TFA-Gain, and TFA-Coherence had good reliability even in the lower quality Groups C and D, of which the WPS-SI had the highest reliability in Groups A and B and the second highest reliability in Groups C and D (figure 4 (b)). Reliability between groups was significantly different for WPS-SI (p = 0.034) and all TFA-derived parameters (table 3; TFA-Gain (p = 0.0007), TFA-Phase (p = 0.002), and TFA-Coherence (p = 0.018)). In the LF band, most CA indices had a good reliability even in lower quality Groups C and D, particularly for WPS-SI (figure 4(c) and table 3). Across all CA indices, there were significant differences in WPS-SI, TFA-Gain and TFA-Coherence between groups (A-D) (table 3).

### Reproducibility between 5-minute Epochs

**Figure 5:**
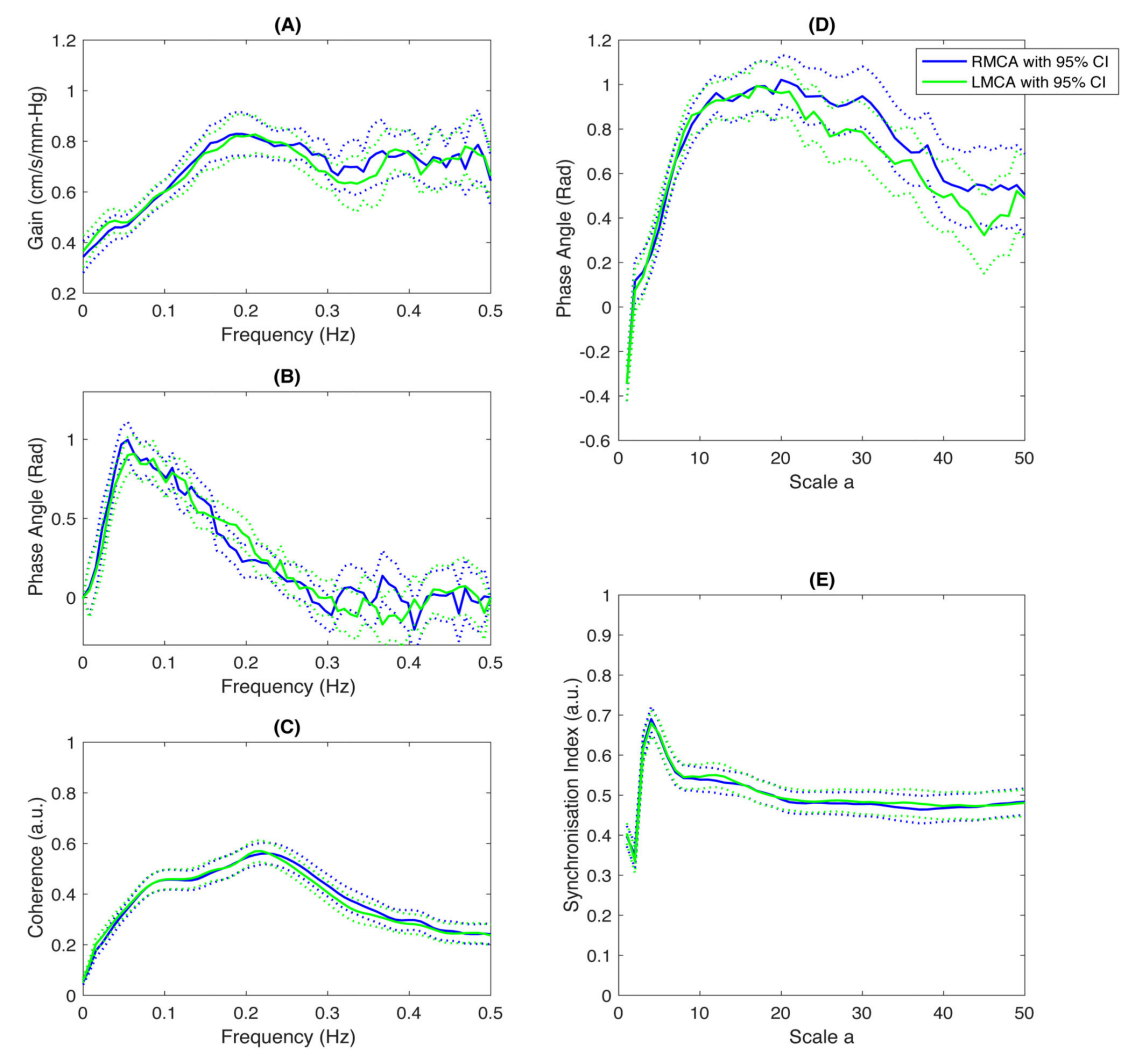
The group-averaged results of frequency-domain indices in the optimal Group A (n=167), showing a consistent trend between MCA sides. (A – C) are TFA-derived indices of Gain, Phase, and Coherence in units of frequency (Hz); and (D – E) are the WPS-derived indices of Phase and Synchronisation Index in units of *wavelet scale a* respectively. Figures are presented as mean (solid lines) with 95% CI (dash lines).

Of 167 / 453 patients with good quality bilateral recordings (Group A), there was good agreement with a consistent trend in CA values between MCA sides (figure 5 and table 4). There were minimal differences in values between 5-min epochs in most CA indices, except for a greater VLF WPS-phase in LMCA in the second 5 minutes of recording (table 4). Across the whole 10 minutes of recording, despite only small differences in the magnitude of CA indices, there was slightly reduced coherence, gain and synchronicity between BP and TCD recordings for some indices across 10-minute recordings compared to each 5-minute epoch, with the expected opposite direction of change in WPS-Phase and TFA-Phase (table 4).

**Figure 6:**
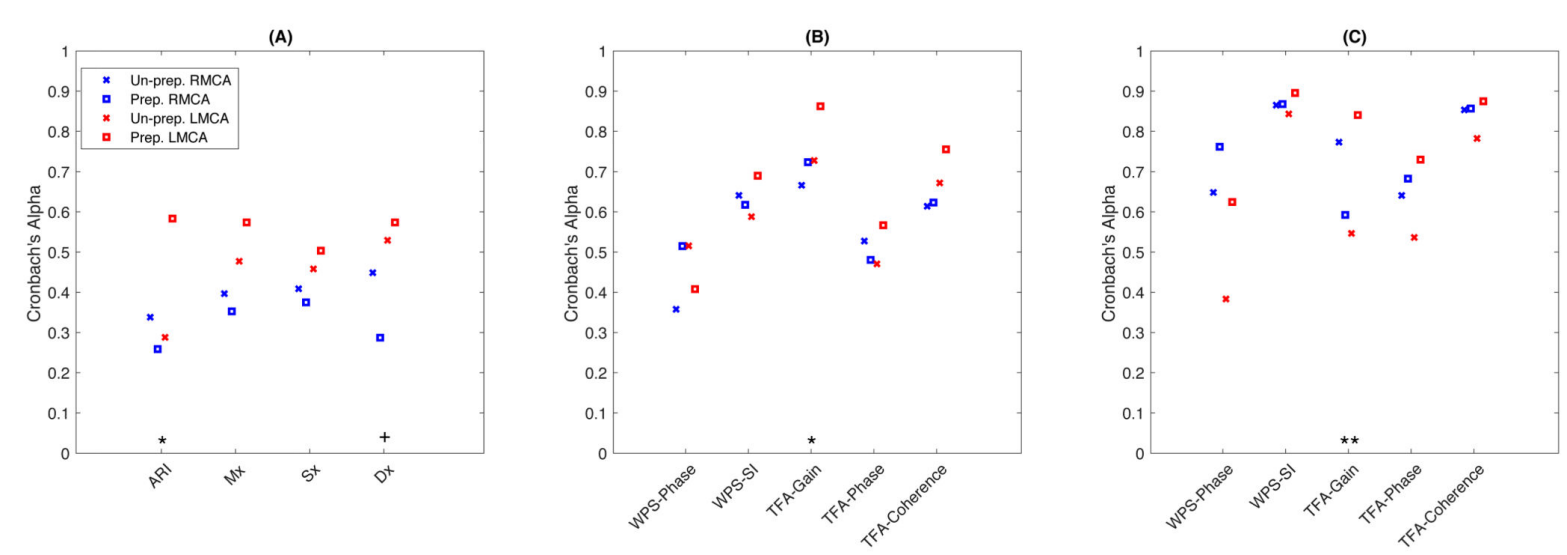
The reproducibility between 5-min epochs in both sides of MCA in the optimal Group A before and after manual data-cleaning (Un-A vs. Group A). (A) time-domain indices; (B) and (C) are frequency-domain indices in VLF and LF bands respectively. Differences of Alpha coefficients were compared between the pre-processed R- and LMCA. Un-Prep., un-processed; Prep., Pre-processed. +, p = 0.06; *, p <0.05; **, p <0.01.

After manual data-processing, reproducibility between 5-min epochs increased in most derived CA indices, especially in LMCA, although time-domain indices in RMCA, WPS-Phase in LMCA, WPS-SI and TFA-Phase in RMCA in VLF band, and TFA-Gain in RMCA in LF band had lower reproducibility after pre-processing (figure 6). Across all CA metrics, reproducibility in LMCA tended to be greater than that in RMCA after pre-processing, but WPS-Phase in both VLF and LF had greater reproducibility in RMCA (figure 6 (b – c)). However, when compared between MCA sides, there were only minimal differences in ARI, Dx and TFA-Gain in both VLF and LF bands (figure 6).

## Discussion

In the large, population-based OXVASC Phenotyped cohort of patients with TIA or minor stroke, intensive data-cleaning, quality assessment and visual inspection of signal quality retained the reliability and increased the reproducibility of derived CA indices. Magnitude of CA indices fell with lowest quality data, with an increased proportion of extreme ARI values, resulting in a non-normal distribution, whilst Pearson’s indices and frequency-domain indices approached zero, despite being normally distributed. However, assessments of CA indices had a reasonable reliability even in lower quality groups and were robust to moderate noise and artefacts, but require careful data pre-processing prior to interpretation in clinical cohorts.

Impaired CA is a marker of poor outcome after major ischaemic stroke (Reinhard et al. 2012; Chi et al. 2018) or intracerebral haemorrhage (Ma et al. 2016; Oeinck et al. 2013) but its physiological relevance, prognostic significance and as a potential target for treatment to reduce the risk of future stroke is unknown, due to small study sizes (Reinhard et al. 2012; Chi et al. 2018), small multi-study datasets (Sanders et al. 2018, 2019), and difficulties in performing physiological challenges in large, frail populations. However, it has great physiological potential to reduce the impact of BP fluctuations on the cerebral circulation (Ma et al. 2019; Peterson et al. 2020) and therefore reduce the risk of stroke and dementia. Recent advances have demonstrated the feasibility of assessment of CA by continuous monitoring of patients at rest, without induced BP changes, whilst CA is beginning to be assessed as an exploratory outcome in ongoing trials assessing drug effects in small vessel disease (e.g. OxHARP and TREAT-SVDs) (Webb et al. 2019; Stevenson et al. 2010). However, the wide range of potential metrics of CA have not been systematically assessed in high risk patients. Given the sensitivity of the non-invasive measurements to artefacts and the reduced quality in longer recordings due to deterioration in ABP signal, patient movement, or physiological changes, methods to improve signal quality, and validation of their reliability, is essential to apply these tests to clinical populations.

Our study demonstrated that the currently favoured index of ARI results in occasional insufficient ARI estimates with extreme values not consistent with other CA indices (e.g. 0 and 9), which have to be excluded from further analysis, resulting in a significant loss of data (Mahdi et al. 2017b). Furthermore, the improved correlations between BP and CBF fluctuation following more manual cleaning indicates that a measured lack of correlation between BP and CBF fluctuations can reflect either intact autoregulation or confounding effects of noise. This is consistent with a recent report of the impact of different levels of added white noise on multiple CA indices derived from artificially generated CBFV signals (Liu et al. 2020), similar to the quality assessment and data-cleaning in our study. Smaller studies also demonstrated failure of the ARI algorithm using simulated noise and lower reliability in problematic CBFV signals (e.g. Group B) (Panerai et al. 2008; Mahdi et al. 2017b; Deegan et al. 2011). As such, appropriate pre-processing is essential, followed by selection of the most robust and reliable CA metrics for future clinical studies in pragmatic populations.

Ours is therefore the first study to determine the effects of manual data-cleaning on the reliability and reproducibility of CA indices in TIA and minor stroke patients. Using CBFV recorded from both sides of MCA, with appropriate pre-processing, resting-state assessments of multiple CA metrics provided sufficiently reliable and reproducible measurements in the majority of patients, even with relatively limited data quality. We may therefore suggest that all studies require automated and blinded, manual pre-processing with quality assessment for analysis of CA, to allow pooling of data for clinical and epidemiological analysis. Furthermore, considering the potentially insufficient ARI estimates, Pearson’s Mx and WPS-SI had greater reliability and reproducibility across all metrics, and would be the most reliable two metrics for future studies determining clinical risk of impaired CA in at-risk populations. One possible explanation of why Mx and WPS-SI perform better than the other metrics is that Pearson’s index is a minute-to-minute method that may not be as sensitive as the frequency-domain indices in detecting short-term variability, whilst the WPS-based indices are derived based on the non-stationary theory, addressing the potential effects caused by non-stationarity of signals (Peng et al. 2010; Sanders et al. 2018, 2019). However, further investigations of impacts of variability and non-stationarity of signals on CA metrics are still warranted.

There are limitations to our study. First, there are limits to categorise artefacts and noise by objective criteria due to a subjective element to any manual cleaning (Panerai et al. 2008; Mahdi et al. 2017b; Deegan et al. 2011), whilst the non-selective, consecutive recruitment of this cohort resulted in unevenly sized quality groups. However, the reduction in CV% with worsening quality indicated the reliability of the quality assessment, and the uneven size of quality groups indicated the objective determination of quality for a given subject. Furthermore, given the large size of our cohort, all quality groups were large enough to be compared statistically. Second, as the cohort was clinically-defined, the aetiology and laterality, particularly of DWI imaging-negative TIAs, could not be reliably determined, preventing assessment of the interaction between site of event and laterality of CA indices. We also consider the potential disagreement of CA between MCA sides, such as might be caused by intracranial stenosis. However, using patients as their own control for comparisons of CA metrics remains the best current way for estimating the internal validity of CA metrics, whilst previous studies have shown that only patients with moderate to severe carotid stenosis or MCA stenosis had significant changes in CA status (Chen et al. 2014; Reinhard et al. 2003; Wang et al. 2015; Payne 2016). As such, the exclusion criterion of severe untreated bilateral carotid stenosis (>70%) used in our study can be regarded as sufficiently rigorous. Thirdly, our tests were performed within the limits of a clinical follow-up assessment in consecutive, unselected patients after TIA or minor stroke. As such, repeated assessments to improve reliability were not possible, participant compliance could not be guaranteed in the context of frailty and mild cognitive impairment and there were a number of elderly patients with difficult temporal bone windows or poor peripheral circulation. As such, there were a greater number of lower quality assessments than may occur in more controlled studies in young, healthy participants. However, this pragmatic study demonstrates the utility of these tests in clinically relevant populations, and demonstrated their relative robustness to moderate noise following appropriate data-cleaning.

Finally, we did not assess the prognostic significance of the different indices, or compare with external clinical characteristics, and therefore cannot provide definitive evidence of the most optimal CA metrics until further follow-up has taken place, ideally in a larger population. Nevertheless, this is the first large study to compare the reliability, reproducibility, and validity of multiple CA metrics within a single elderly population at an increased risk of stroke or TIA.

## Conclusions

Appropriate pre-processing produces reliable, consistent indices of cerebral autoregulation from adequate quality, resting-state recordings. Assessments of cerebrovascular autoregulation are valid and robust to moderate noise and artefact, with reliability and reproducibility being greatest for Mx and WPS-SI. This provides a robust basis to investigate the relationship between cerebral autoregulation, clinical characteristics and the risk of recurrent stroke and dementia in high-risk patients.

## Supplementary Material

Supplementary material

## Figures and Tables

**Table 1:** Basic population characteristics.

	All (n=453)	Group A (n=167)	Group B (n=65)	Group C (n=25)	Group D (n=42)	Group E (n=154)	p
Age, yr	65.4 ± 13.6	62.3 ± 14.3	63.2 ± 13.1	68.6 ± 15.1	67.6 ± 13.3	68.7 ± 12.0	0.0002
Female	177 (39.1)	60 (35.9)	26 (40.0)	11 (44.0)	14 (33.3)	66 (42.9)	0.55
Hypertension	194 (42.8)	59 (35.3)	29 (44.6)	12 (48.0)	19 (45.2)	75 (48.7)	0.17
Diabetes Mellitus	43 (9.5)	13 (7.8)	8 (12.3)	2 (8.0)	6 (14.3)	14 (9.1)	0.67

Values are numbers (percentage) and mean ± SD.

**Table 2:** The categorised groups and variability of signals in each quality group.

Groups (n)	CV% of MABP	CV% of MFv	
		RMCA	LMCA
Un-A (raw data; n=166)	4.72 (3.98 – 5.46)	7.53 (6.11 – 8.94)	7.78 (6.96 – 8.60)
Group A (n=167)	4.05 (3.79 – 4.31)^x^	6.33 (5.34 – 7.32)	5.62 (5.27 – 5.98)***
Group B (n=65)	4.14 (3.73 – 4.55)	6.80 (5.37 – 8.23)	11.7 (-0.07 – 23.4)
Group C (n=25)	7.05 (5.56 – 8.53)	5.12 (3.92 – 6.33)	5.02 (3.86 – 6.17)
Group D (n=42)	5.53 (4.51 – 6.55)	5.86 (5.14 – 6.57)	6.30 (5.35 – 7.25)
Group E (n=154)	4.95 (4.50 – 5.40)^‡^	**No Data Side**	**Signal Side**
		61.8 (48.5 – 75.2)^‡^	12.5 (7.9 – 17.1)^†^

Un-A: Median-filtered but no manual cleaning; Group A: All optimal in both ABP and CBFV; Group B: Either side or both sides of CBFV are adequate; Group C: ABP is adequate or unusable and CBFV is optimal; Group D: ABP is adequate or unusable and CBFV is optimal or adequate; and Group E: ABP is optimal, adequate, or unusable, one side of CBFV is optimal or adequate but the other side of CBFV is unusable (i.e. quality = 1: severe artefact) or no data (i.e. quality = 0; the measured signal is noise, with no usable information of CBFV being measured). Data are presented as mean (95% Confidence Interval). MABP, mean arterial blood pressure; MFv, mean cerebral blood flow velocity; CV%, percentage of coefficient of variation;

x p = 0.069;

*** *p* <0.0001 (for Un-A vs. Group A, by paired sample t-test).

† p-trend <0.05;

‡ *p-trend* <0.0001 for Group A–E.

Two patients with only left-sided window are in Groups A and B respectively.

**Table 3:** Cronbach’s alpha between right and left MCA in each signal quality group.

	Un-A	A*	B	C	D	E	p-value^†^
**Time-Domain Indices**							
ARI	0.68	0.68	0.73	0.87	0.57	-0.11	0.19
Mx	0.79	0.77	0.73	0.76	0.79	0.02	0.98
Sx	0.78	0.75	0.64	0.78	0.81	0.19	0.70
Dx	0.81	0.82	0.80	0.72	0.80	0.24	0.86
**Grand Mean**	0.77	0.76	0.73	0.78	0.74	0.09	N/A
**Frequency-Domain Indices in VLF**							
WPS-Phase	0.77	0.66	0.50	0.76	0.67	0.12	0.62
WPS-Sync. Index	0.84	0.85	0.87	0.97	0.92	0.15	0.034
TFA-Gain	0.84	0.84	0.76	0.98	0.91	0.18	0.0007
TFA-Phase	0.69	0.68	0.42	0.93	0.67	-0.16	0.002
TFA-Coherence	0.88	0.82	0.82	0.91	0.96	-0.14	0.018
**Grand Mean**	0.80	0.77	0.67	0.93	0.83	0.03	N/A
**Frequency-Domain Indices in LF**							
WPS-Phase	0.82	0.66^‡^	0.78	0.93	0.86	-0.60	0.06
WPS-Sync. Index	0.90	0.84	0.75	0.95	0.91	-0.63	0.036
TFA-Gain	0.70	0.78	0.77	0.94	0.78	0.04	0.02
TFA-Phase	0.59	0.84^§^	0.70	0.89	0.76	-0.43	0.27
TFA-Coherence	0.85	0.81	0.42	0.96	0.87	-0.50	0.0008
**Grand Mean**	0.77	0.79	0.68	0.93	0.84	-0.42	N/A

Values were approximated to two significant numbers for clarity.

* Statistical comparisons between Group A and Un-A were performed.

‡ p < 0.05;

§ p <0.001.

† Comparisons are only between Groups A, B, C, and D because of poor reliability in Group E.

**Table 4:** The group-averaged results of all CA indices of the optimal quality Group A.

(n=167)	RMCA			LMCA		
	10-min	1^st^ 5-min	2^nd^ 5-min	10-min	1^st^ 5-min	2^nd^ 5-min
**Time-Domain Indices**						
ARI	5.1 (3.9 – 6.23)	5.0 (3.40 – 6.53)	4.8 (3.6 – 7.0)	4.5 (3.3 – 6.1)	4.6 (3.2 – 6.4)	4.6 (3.2 – 6.6)
Mx	0.27 (0.16 – 0.44)	0.33 (0.15 – 0.46)	0.34 (0.16 – 0.51)*	0.32 (0.16 – 0.49)	0.38 (0.15 – 0.50)	0.38 (0.20 – 0.53)*
Sx	0.17 (0.06 – 0.31)	0.21 (0.01 – 0.34)	0.22 (0.04 – 0.38)	0.22 (0.08 – 0.37)	0.22 (0.06 – 0.37)	0.26 (0.07 – 0.40)
Dx	0.21 (0.11 – 0.38)	0.26 (0.08 – 0.41)	0.26 (0.09 – 0.42)*	0.26 (0.09 – 0.40)	0.27 (0.11 – 0.41)	0.28 (0.10 – 0.44)
**Time-Domain Indices**						
**WPS-Phase**						
VLF	0.89 (0.43 – 1.23)	0.74 (0.30 – 1.13)	0.72 (0.39 – 1.23)	0.74 (0.37 – 1.06)	0.68 (0.24 – 1.09)	0.77 (0.27 – 1.15)^†^
LF	0.78 (0.55 – 1.01)	0.77 (0.56 – 0.96)	0.77 (0.55 – 1.06)	0.79 (0.57 – 1.04)	0.76 (0.56 – 1.01)	0.75 (0.53 – 1.08)
**WPS-SI**						
VLF	0.47 (0.38 – 0.59)	0.51 (0.40 – 0.63)***	0.54 (0.44 – 0.65)^#^	0.48 (0.37 – 0.61)	0.51 (0.41 – 0.63)^#^	0.56 (0.42 – 0.66)^#^
LF	0.54 (0.43 – 0.68)	0.60 (0.45 – 0.71)**	0.56 (0.42 – 0.70)^§^	0.59 (0.43 – 0.70)	0.60 (0.44 – 0.72)**	0.58 (0.45 – 0.69)
**TFA-Gain**						
VLF	0.46 (0.29 – 0.61)	0.48 (0.34 – 0.68)^x^	0.47 (0.31 – 0.65)	0.43 (0.33 – 0.61)	0.49 (0.32 – 0.67)***	0.45 (0.33 – 0.68)
LF	0.68 (0.45 – 0.91)	0.71 (0.50 – 0.98)*	0.71 (0.51 – 0.97)*	0.68 (0.49 – 0.89)	0.74 (0.55 – 0.96)**	0.71 (0.52 – 0.94)
**TFA-Phase**						
VLF	0.87 (0.56 – 1.16)	0.83 (0.46 – 1.17)	0.85 (0.44 – 1.24)	0.81 (0.52 – 1.13)	0.82 (0.42 – 1.21)	0.80 (0.40 – 1.13)
LF	0.55 (0.36 – 0.85)	0.57 (0.34 – 0.83)	0.53 (0.30 – 0.81)	0.57 (0.39 – 0.88)	0.56 (0.32 – 0.85)	0.62 (0.36 – 0.90)
**TFA-Coherence**						
VLF	0.31 (0.20 – 0.42)	0.34 (0.24 – 0.48)**	0.35 (0.25 – 0.48)***	0.33 (0.23 – 0.44)	0.35 (0.23 – 0.50)***	0.39 (0.25 – 0.47)^#^
LF	0.47 (0.29 – 0.65)	0.51 (0.37 – 0.68)^#^	0.51 (0.33 – 0.68)***,†	0.47 (0.32 – 0.67)	0.55 (0.36 – 0.72)^#^	0.52 (0.36 – 0.69)**

Data were presented as median and (IQR). R- and LMCA, right- and left middle cerebral artery; VLF, very low frequency; LF, low frequency.

Comparisons between 5-min and 10-min:

x p = 0.054;

* p <0.05;

** p <0.01;

*** p <0.001;

# p <0.0001.

Comparisons between the two 5-min epochs:

† p <0.05;

§ p <0.01.
