# Supplementary material for: Reliability, reproducibility and validity of dynamic cerebral autoregulation in a large cohort with transient ischaemic attack or minor stroke
Source: Physiol Meas. Author manuscript; Available in PMC 2021 Jan 13. (PMC7116588; doi:10.1088/1361-6579/abad49)
Supplement: Supplementary material [file EMS95128-supplement-Supplementary_material.pdf]

Supplemental Materials

Reliability, Reproducibility and Validity of Dynamic Cerebral Autoregulation in a Large Cohort with Transient Ischaemic Attack or Minor Stroke

Yun-Kai Lee, Peter M. Rothwell, Stephen J. Payne*, Alastair J.S. Webb*

Content:

Details of Calculations of Dynamic Cerebral Autoregulation Indices ---------------------------p.2

Regression between ARI and Mx before and after removing erroneous ARI 0 and 9-------p.5

Associations between Mx and frequency-domain indices in the optimal Group A -----------p.6

The study flowchart. --p.7

The proportion of the labelled signal quality in each group, demonstrating an increased proportion of noise and artefacts in worse quality group ---p.8

The distribution of Pearson's correlation coefficient indices of Sx and Dx in both sides of MCA---p.9

A. Details of Calculations of Dynamic Cerebral Autoregulation Indices

1. Time-Domain Analysis Methods

1.1 Autoregulatory Index (ARI)

ARI is a gauging system that commonly being applied to assess the status of CA by producing a sudden drop in ABP and further quantify how fast the response of CBFV to change in ABP (Mahdi et al. 2017). It is graded to 10 levels (from 0 – 9), where ARI = 9 indicates that CBFV returns to baseline rapidly, indicating an effective status of CA, and ARI = 0 represents a lack of response of CBFV to ABP, meaning a completely impaired status, respectively.

ARI	*T*	*D*	*K*
0	2	0	0
1	2	1.60	0.20
2	2	1.50	0.40
3	2	1.15	0.60
4	2	0.9	0.80
5	1.9	0.75	0.90
6	1.6	0.65	0.94
7	1.2	0.55	0.96
8	0.87	0.52	0.97
9	0.65	0.50	0.98

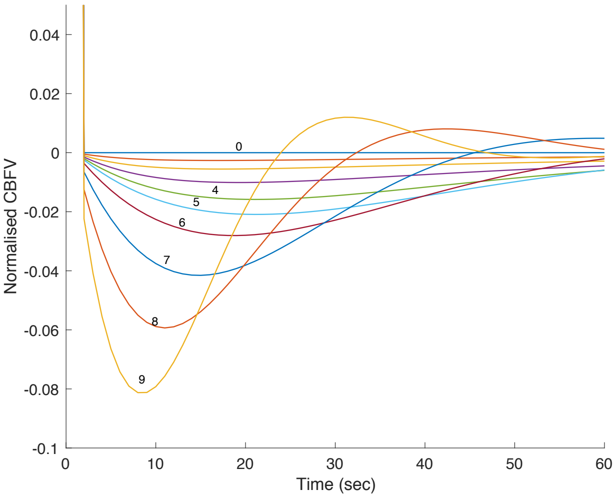

Table A1 and Figure AI. The table of the defined parameters of T (time constant), D (damping factor), and K (gain parameter), and the corresponding ARI model.

1.2 Pearson's Correlation Coefficient-Based Indices (Mx, Sx, and Dx)

Based on the principle that intact status of CA will have lower association between changes in ABP and CBFV, the correlation coefficient will thus tend to have lower values. The mean flow index (Mx) is derived by calculating the Pearson's correlation coefficient between the mean ABP and mean CBFV, calculated as follows:

$$r = \frac{\sum_i (a_i - \bar{a})(b_i - \bar{b})}{\sqrt{\sum_i (a_i - \bar{b})^2}\sqrt{\sum_i (a_i - \bar{b})^2}} \qquad (Eq. A1)$$

where a_i and b_i are the input signals of beat-to-beat ABP and CBFV in time series, and $\bar{a}$ and $\bar{b}$ are the mean of the input signals, respectively. The Sx and Dx indices are derived by the same calculations except for replacing the input signal of mean ABP with systolic and diastolic components of ABP. Since Pearson's correlation coefficient has a standardised value ranging between -1 and 1, it can be assessed as a time-domain index reflecting the dynamic changes in the cerebral vasculature, and a higher value of Mx (Sx and Dx) indicates a more passive dependence of CBFV on ABP, thereby represents an impaired status of CA (Mahdi et al. 2017; Xiong et al. 2017; Donnelly et al. 2017; Liu et al. 2017); Czosnyka et al. 2001).

2. Frequency-Domain Analysis Methods

2.1 Transfer Function Analysis (TFA)

Computed indices of CA can also be derived by using the fast Fourier transform (FFT). In this study, we applied TFA method based on Welch's method using a 128-sec Hanning window with 50% overlap to assess the relationship between the input (beat-to-beat ABP) and output signals of CBFV as described in previous studies (Claassen et al. 2015; Latka et al. 2005; Peng et al. 2010), where the transfer function $H_{PV}(f)$ between ABP and CBFV of MCA was expressed as follows:

$$H_{PV}(f) = \frac{S_{PV}(f)}{S_{PP}(f)} \quad (Eq. A2)$$

where $S_{PV}(f)$ is the cross-spectrum between the ABP and CBFV and $S_{PP}(f)$ is the autospectrum of ABP signal (Claassen et al. 2015; Latka 2005; Peng et al. 2010). The TFA-based coherence is calculated as:

$$Coherence: \quad Coh(f) = \frac{|S_{PV}(f)|^2}{[S_{PP}(f) S_{VV}(f)]} \quad (Eq. A3)$$

where $S_{VV}(f)$ represents the autospectrum of CBFV signal. The TFA-derived magnitude (gain) and phase of the complex transfer function $H_{PV}(f)$ can also be calculated from the real (H_R) and imagine (H_I) parts, respectively, expressed as the following equations:

$$Gain: \quad |H_{PV}(f)| = [\, {H_I}^2 + {H_R}^2]^{\frac{1}{2}} \quad (Eq. A4)$$

$$Phase: \quad \psi_{PV}(f) = \tan^{-1}\left[\frac{H_I(f)}{H_R(f)}\right] \quad (Eq. A5)$$

In the present study, we determined the mean values of TFA-derived parameters in the most active frequency bands of VLF (0.02 – 0.07 Hz) and LF (0.07 – 0.2 Hz) calculated (Claassen et al. 2015).

2.2 Wavelet Phase Synchronisation (WPS)

The WPS method overcomes the limitations of non-stationarity of signals. In brief, it stretches and scales the mother wavelet and calculate the similarity of the corresponding signal area until the whole signal is covered. The complex continuous wavelet transform of a time-series signal $x(t)$ is described as follows:

$$W_x(a,t) = \frac{1}{\sqrt{a}} \int_{-\infty}^{\infty} x(t) \psi * \left(\frac{t - t_0}{a}\right) dt \quad (Eq.\ A6)$$

where $W_x(a, t)$ is the derived wavelet coefficient, a function of wavelet scale *a* and time scale *t*, and the selected 'Morlet' mother wavelet is defined as:

$$\psi(t) = \frac{1}{\sqrt{\pi f_b}} e^{2\pi i f_c t} e^{-t^2/f_b} \quad (Eq.\ A7)$$

where the centre frequency and bandwidth are denoted by f_c and f_b respectively [7, 8]. Furthermore, the relationship between the *wavelet scale a* and the pseudo-frequency *f (Hz)* can be converted by calculating the reciprocal as follows:

$$f = \frac{f_c}{a \delta t} \quad (Eq.\ A8)$$

where δt represent the centre period (Latka et al. 2005; Peng et al. 2010). Hence, the defined frequency bands from the previous consensus papers (Claassen et al. 2015) correspond to wavelet scale *a* at VLF [scale 14−50] and LF bands [scale 5−14].

The instantaneous phase can be calculated from the wavelet coefficient W_x using the following equation:

$$\phi_x(a, t) = \angle W_x = -i \log\left[\frac{W_x(a,t)}{|W_x(a,t)|}\right] \quad (Eq.\ A9)$$

Hence, instantaneous phase of ABP and CBFV, denoted as $\phi_P(a,t)$ and $\phi_V(a,t)$, respectively, can all be calculated from the wavelet coefficients of $W_P(a,t)$ and $W_V(a,t)$, respectively. The WPS-derived phase shift between ABP and CBFV is calculated as:

$$\Delta\phi_{PV}(a,t) = \phi_P(a,t) - \phi_V(a,t) \quad (Eq.\ A10)$$

whilst its circular mean, $\Delta\bar{\phi}_{PV}$, is then calculated, in order to estimate the phase dynamics as well as the synchronisation behaviour, as follows:

$$Phase: \quad \Delta\bar{\phi}_{PV}(a) = \tan^{-1}\left\{\frac{\sum_t \sin(\Delta\phi_{PV}(a,t))}{\sum_t \cos(\Delta\phi_{PV}(a,t))}\right\} \quad (Eq. A11)$$

According to previous studies, the WPS-derived synchronisation index (WPS-SI) (γ) can be calculated using:

$$Sync. Index: \quad \gamma(a) = \frac{1}{N}\left(\left[\sum_t \sin(\Delta\phi_{PV}(a,t))\right]^2 + \left[\sum_t \cos(\Delta\phi_{PV}(a,t))\right]^2\right)$$

$$(Eq. A12)$$

where N is the data length of the signal (Latka et al. 2005; Peng et al. 2010). WPS-SI is ranging between 0 and 1, and a higher value represents a lower variation over time, indicating the phase shifts between signals have lower variation over time.

3. Cronbach's Alpha

Cronbach's Alpha is a measure of reliability, estimating how closely related a set of items are, based on the internal consistency. The calculation of Cronbach's Alpha (Cronbach (1951); Diedenhofen & Musch (2016)) is presented as follows:

$$\alpha = \frac{N\bar{c}}{\bar{v} + (N-1)\bar{c}} \qquad (Eq. A13)$$

where N represents the number of items, $\bar{c}$ is the mean inter-item covariance, and $\bar{v}$ is the mean variance. Theoretically, Cronbach's Alpha is ranging between 0 and 1 and a higher value indicates that there is a more reliable result. However, the calculation of Cronbach's Alpha can be negative in practice due to the negative average covariance among the items (i.e. sum of the item variance is larger), in which case, the estimate should be treated as very un-reliable.

B. Supplemental Results

Supplementary table I. Regression between ARI and Mx before and after removing ARI 0 and 9.

	Before Removing ARI 0 and 9						After Removing ARI 0 and 9						
	RMCA			LMCA				RMCA			LMCA		
**Group (n)**	r^2	p	β	r^2	p	β	**(n)**	r^2	p	β	r^2	p	β
**Group A (n=167)**	0.49	$<10^{-25}$	-0.067	0.29	$<10^{-12}$	-0.054	R:152; L:148	0.55	$<10^{-26}$	-0.09	0.53	$<10^{-25}$	-0.09
**Group B (n=65)**	0.59	$<10^{-12}$	-0.07	0.40	$<10^{-7}$	-0.06	R: 59; L: 59	0.59	$<10^{-11}$	-0.08	0.47	$<10^{-8}$	-0.08
**Group C (n=25)**	0.15	0.053	-0.034	0.11	0.11	-0.026	R: 21; L: 22	0.77	$<10^{-6}$	-0.10	0.56	$<10^{-4}$	-0.065
**Group D (n=42)**	0.13	0.02	-0.035	0.008	0.58	-0.009	R: 34; L: 31	0.68	$<10^{-8}$	-0.11	0.59	$<10^{-6}$	-0.11
**Group E No Data Side (n=154)**	0.013	0.17	-0.008		N / A		n = 89	0.07	0.001	-0.025		N / A	

Supplementary table II. Associations between Mx and frequency-domain indices in the optimal Group A.

Comparisons (n=167)	RMCA r^2	RMCA p	RMCA β	LMCA r^2	LMCA p	LMCA β
**Mx vs. TFA-Gain**						
VLF	0.06	0.0015	0.30	0.05	0.0043	0.26
LF	0.002	0.57	0.08	0.0006	0.76	0.032
**Mx vs. TFA-Phase**						
VLF	0.03	0.019	-0.52	0.006	0.32	-0.21
LF	0.02	0.073	-0.31	0.011	0.19	-0.19
**Mx vs. TFA-Coherence**						
VLF	0.30	<0.0001	0.40	0.24	<0.0001	0.34
LF	0.06	0.0024	0.27	0.074	0.0004	0.26
**Mx vs. WPS-Phase**						
VLF	0.03	0.026	-0.52	0.009	0.21	-0.27
LF	0.0042	0.41	-0.15	0.01	0.20	-0.18
**Mx vs. WPS-SI**						
VLF	0.20	<0.0001	0.32	0.19	<0.0001	0.28
LF	0.10	<0.0001	0.28	0.07	0.00041	0.21

Supplementary figure I. The study flowchart. TCD, transcranial Doppler.

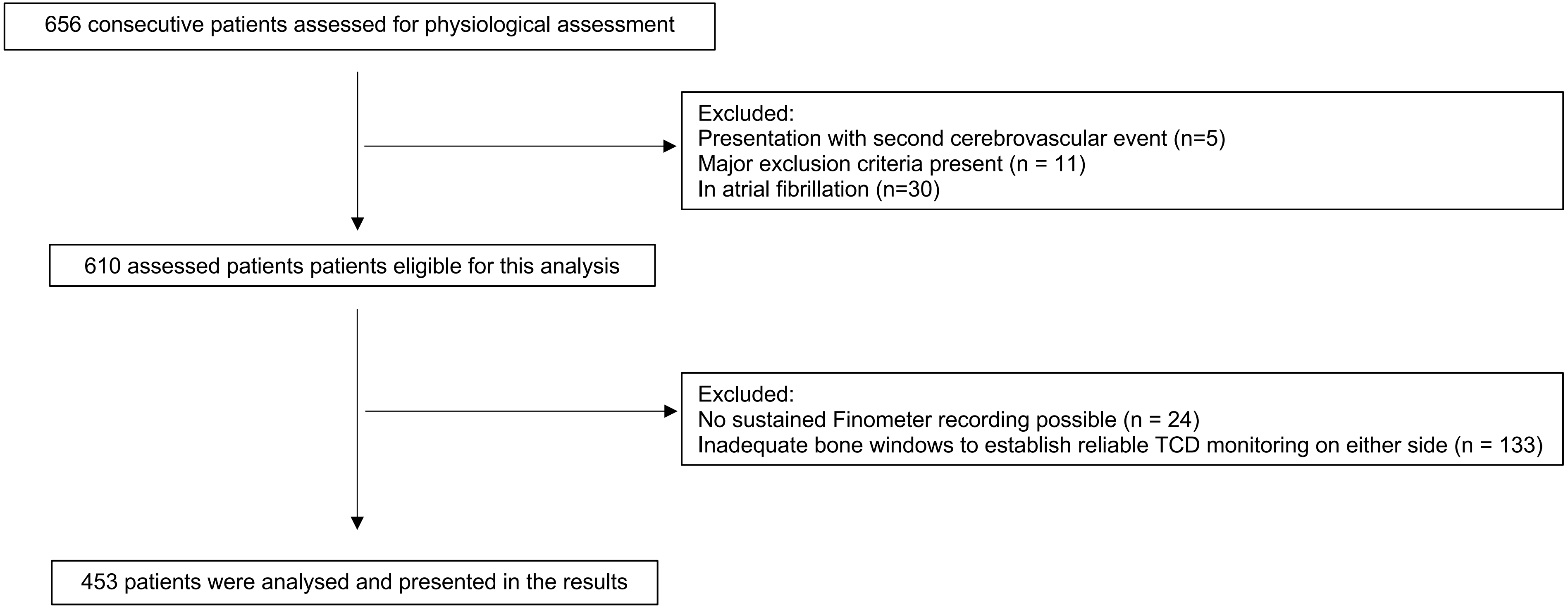

Supplementary figure II. The proportion of the labelled signal quality in each group, demonstrating an increased proportion of noise and artefacts in worse quality group. (A) The ABP signal; (B) and (C) are CBFV in R- and LMCA, respectively. 3, clean; 2, adequate but problematic; 1, severe artefact; and 0, no data.

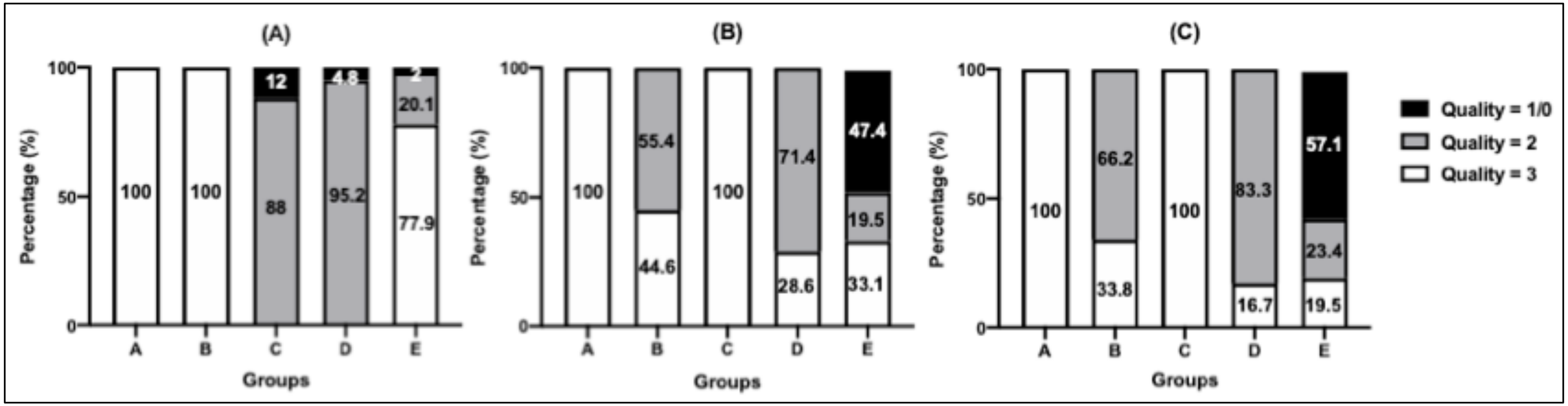

Supplementary figure III. The distribution of Pearson's correlation coefficient indices of Sx and Dx in both sides of MCA. (A – B) are Sx in R- and LMCA; and (C – D) show Dx in R- and LMCA, respectively. *, p <0.05; **, p <0.01; and †, p <0.0001.

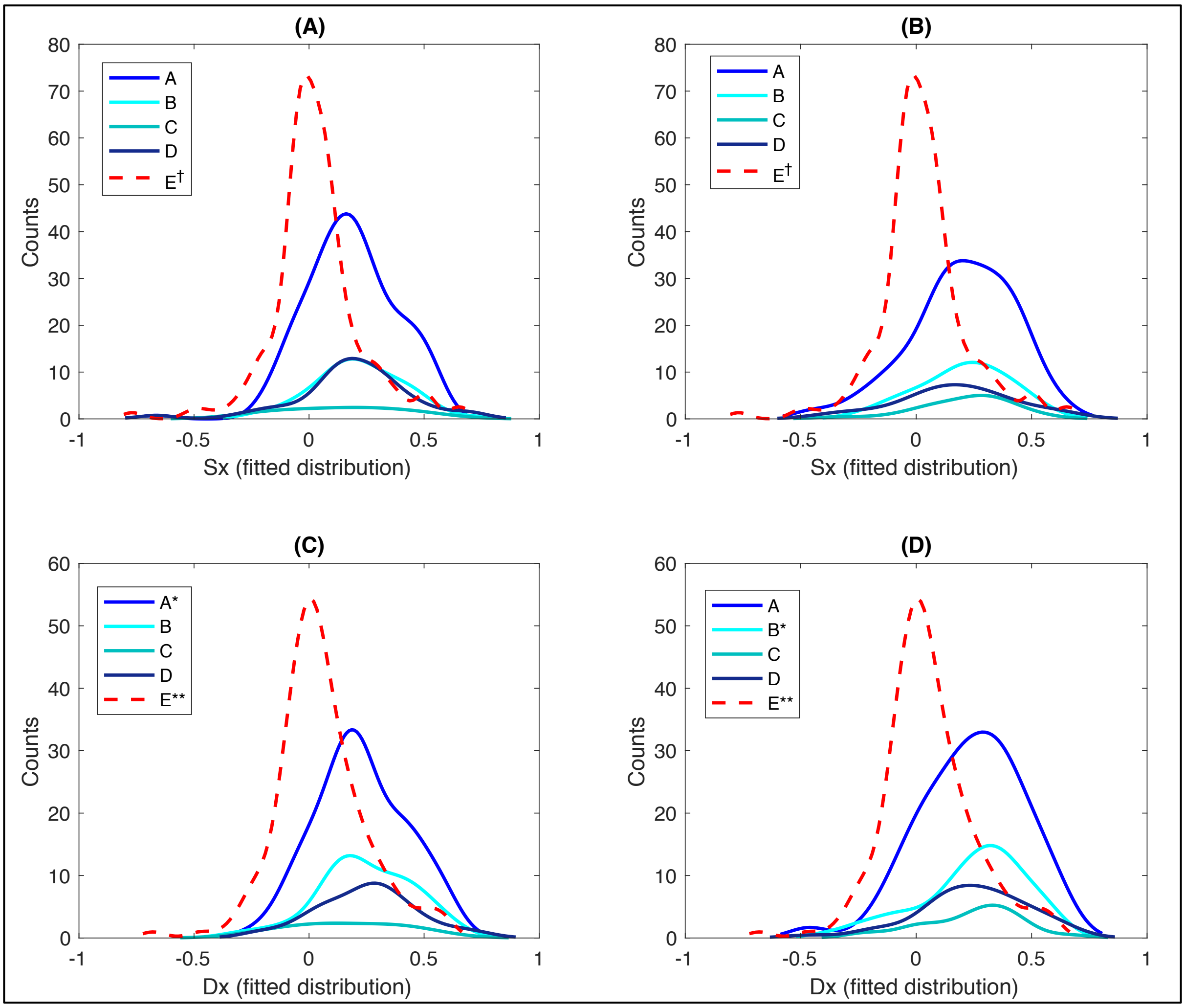

Supplementary Reference

Claassen J A, Meel-Van Den Abeelen A S, Simpson D M, and Panerai R B 2015 Transfer function analysis of dynamic cerebral autoregulation: A white paper from the International Cerebral Autoregulation Research Network. *J Cereb Blood Flow Metab* 36:665–680

Cronbach L J 1951 Coefficient alpha and the internal structure of tests. *Psychometrika* 16:297–334.

Czosnyka M, Smielewski P, Piechnik S, Steiner L A, and Pickard J D 2001 Cerebral autoregulation following head injury. *J Neurosurg* 95:756-763

Diedenhofen B, and Musch J 2016 cocron : A Web Interface and R Package for the Statistical Comparison of Cronbach's Alpha Coefficients. *Int J Internet Sci* 11:51–60.

Donnelly J, Budohoski K P, Smielewski P, and Czosnyka M 2016 Regulation of the cerebral circulation: Bedside assessment and clinical implications. *Crit Care* 20:1–17

Latka M, Turalska M, Glaubic-Latka M, Kolodziej W, Latka D, and West B J 2005 Phase dynamics in cerebral autoregulation. *Am J Physiol Circ Physiol* 289:H2272–H2279

Liu X, Donnelly J, Czosnyka M, Aries M J H, Brady K, Cardim D, Robba C, Cabeleira M, Kim DJ, Haubrich C, Hutchinson PJ, and Smielewski P 2017 Cerebrovascular pressure reactivity monitoring using wavelet analysis in traumatic brain injury patients: A retrospective study. *PLoS Med* 14:1–19

Mahdi A, Nikolic D, Birch A A, Olufsen M S, Panerai R B, Simpson D M, and Payne S J 2017a Increased blood pressure variability upon standing up improves reproducibility of cerebral autoregulation indices. *Med Eng Phys* 47:151–158

Peng T, Rowley A B, Ainslie P N, Poulin M J, and Payne S J 2010. Wavelet phase synchronization analysis of cerebral blood flow autoregulation. *IEEE Trans Biomed Eng* 57:960–968

Xiong L, Liu X, Shang T, Smielewski P, Donnelly J, Guo Z N, Yang Y, Leung T, Czosnyka M, Zhang R, Liu J, and Wong K S 2017 Impaired cerebral autoregulation: Measurement and application to stroke. *J Neurol Neurosurg Psychiatry* 88:520–531
